# Fulminant Mucormycosis Involving Paranasal Sinuses: A Rare Case Report

**DOI:** 10.1155/2014/465919

**Published:** 2014-01-09

**Authors:** Komali Garlapati, Sunanda Chavva, Rahul Marshal Vaddeswarupu, Jyotsna Surampudi

**Affiliations:** ^1^Department of Oral Medicine and Radiology, Room No. 1, Panineeya Mahavidyalaya Institute of Dental Sciences and Research Centre, Road No. 5, Kamalanagar, Dilsukhnagar, Hyderabad, Andhra Pradesh 500060, India; ^2^Department of Oral Medicine and Radiology, Panineeya Mahavidyalaya Institute of Dental Sciences and Research Centre, Hyderabad, Andhra Pradesh 500060, India

## Abstract

Mucormycosis is an opportunistic fulminant fungal infection, which has the ability to cause significant morbidity and frequently mortality in the susceptible patient. Common predisposing factors include diabetes mellitus and immunosuppression. The infection begins in the nose and paranasal sinuses due to inhalation of fungal spores. The fungus invades the arteries leading to thrombosis that subsequently causes necrosis of the tissue. The infection can spread to orbital and intracranial structures either by direct invasion or through the blood vessels. Here we describe a case of mucormycosis of maxillary antrum extending to ethmoidal and frontal sinus and also causing necrosis of left maxilla in an uncontrolled diabetic individual to emphasize early diagnosis and treatment of this fatal fungal infection.

## 1. Introduction

Mucormycosis (phycomycosis, zygomycosis) is a rare opportunistic infection caused by fungi belonging to the Mucorales order and the Mucoraceae family [1]. Mucormycosis was first described by Paultauf in 1885 [1]. It is recognized as one of the most rapidly progressive lethal form of fungal infection in human beings with a high mortality of 70–100% [2]. The most commonly reported form of the disease is rhinocerebral mucormycosis, which is characterized by progressive fungal invasion of the hard palate, paranasal sinuses, orbit, and brain [3]. It can be subdivided into rhinomaxillary and rhino-oculocerebral forms, the latter being characterized by a high mortality rate [1]. The conditions predisposing to mucormycosis are diabetes mellitus, malnutrition, haematological malignancies, neutropenia, burns, surgical procedures, occlusive dressings, antibiotics, long-term steroid therapy, and immunosuppressive therapy [2]. Successful management of this fulminant infection requires early recognition of the disease and aggressive medical and surgical interventions to prevent the high morbidity and mortality associated with the disease process [3].

We report a case of mucormycosis causing maxillary necrosis with involvement of left side maxillary, ethmoidal, and frontal sinuses.

## 2. Case Report

**Figure 1:**
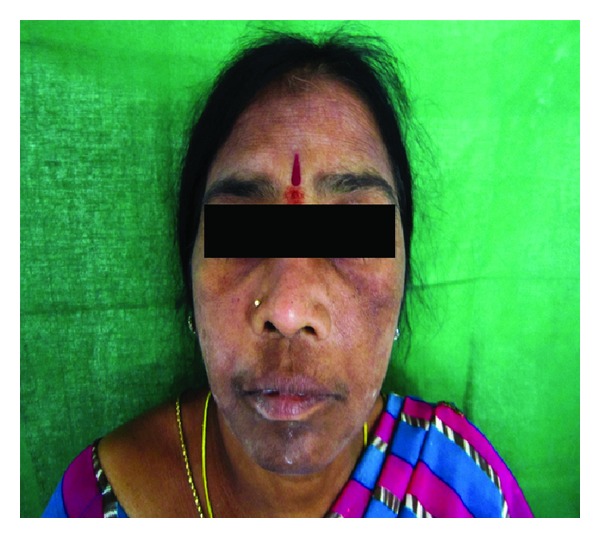
Extraoral examination reveals swelling in the left side of the face just below the eye.

**Figure 2:**
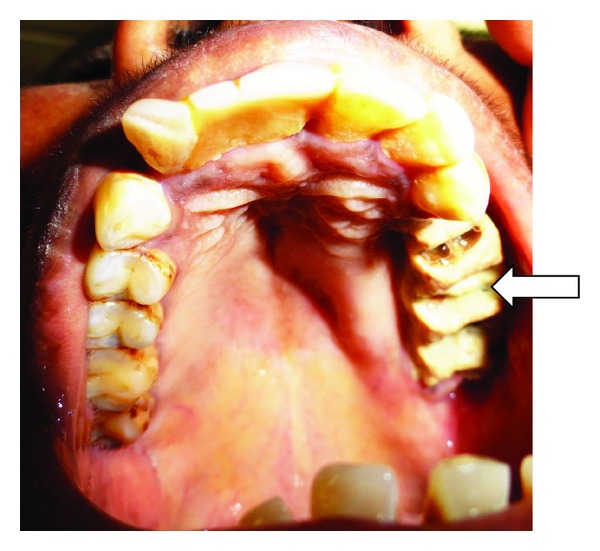
Intraoral examination reveals necrotic bone with pus discharge in relation to left maxilla (white arrow).

A 40-year-old female patient presented with dull pain and purulent discharge in left posterior maxillary teeth region since 6 months following the extraction of teeth (24, 25, 26, 27, and 28). She presents history of dull aching pain with intermittent extra oral swelling over left maxilla and numbness of left side of upper lip. Patient had visited general physician with facial cellulitis 6 months ago and was diagnosed with uncontrolled type II diabetes mellitus (fasting blood sugar level: 300 mg/dL, postprandial blood sugar level: 402 mg/dL) and had taken treatment for the same. During the treatment, since the patient complained of pain and mobility of left maxillary posterior teeth, she was referred for the extraction of the 24, 25, 26, 27 and 28. Vital signs were within normal limits. Extraorally, a mild swelling was observed over left side of middle third of the face causing obliteration of nasolabial fold,there was discolouration of overlying skin and periorbital oedema of left eye was also observed (Figure 1). Intraoral examination revealed, necrotic alveolar bone with evidence of foul discharge and tenderness in relation to maxillary left edentulous region (Figure 2). Based upon the clinical findings, differential diagnoses of deep fungal infection, avascular necrosis of the maxillary bone were considered.

**Figure 3:**
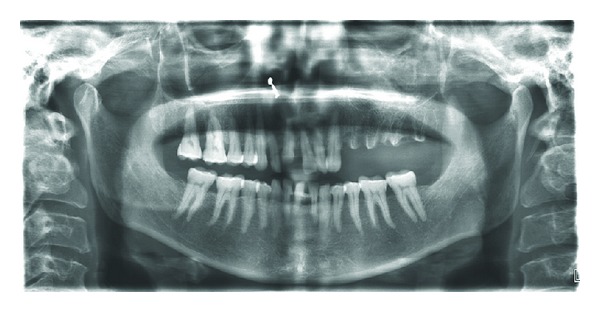
Orthopantomogram reveals missing teeth (24, 25, 26, 27,  and  28) with unhealed sockets.

**Figure 4:**
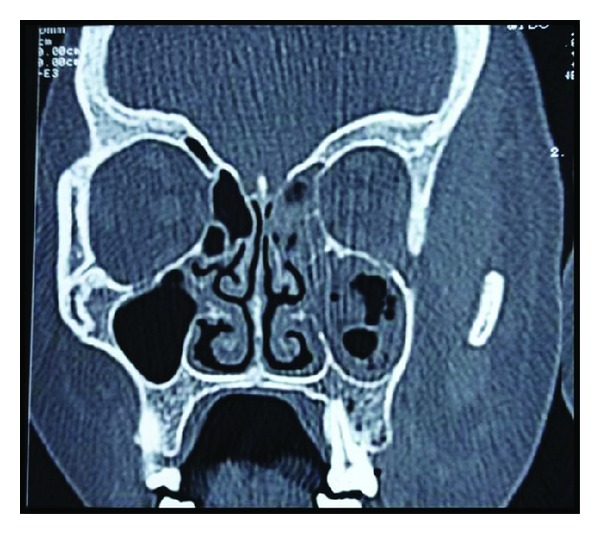
Coronal view of CT showing involvement of left maxillary sinus, nasal conchae, and ethmoidal sinus extending up to frontal sinus.

**Figure 5:**
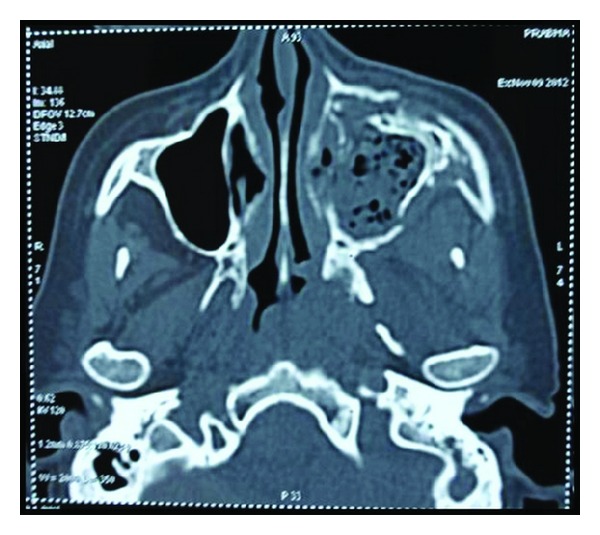
Axial view of CT showing destruction of posterior, medial, and anterior walls of left maxillary sinus.

Radiographic examination revealed opacification of left maxillary sinus (Figure 3). CT scan revealed nonhomogenous opacification of left maxillary sinus causing obstruction of left osteomeatal unit extending into middle meatus, ethmoidal, and frontal sinus (Figure 4) causing destruction of walls of left maxillary and ethmoidal sinuses (Figure 5).

**Figure 6:**
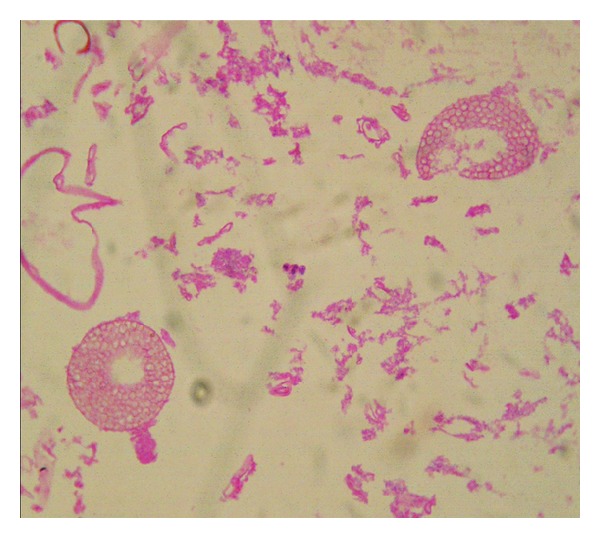
Histopathology of biopsied tissue showing nonseptate obtuse angled fungal hyphae and sporangiophores with spores.

On histological examination of biopsied specimen, necrosed tissue with fungal hyphae which were nonseptate obtuse angles, sporangiophores with spores present (Figure 6) suggestive of mucormycosis.

Immediately blood sugar levels were controlled with insulin and the necrotic bone of maxillary left edentulous region was removed under local anesthesia. The patient was advised and administered amphotericin-B 1 mg/kg body weight/day intravenously, slow infusion over 4–6 hours for 2 weeks, after a test dose of 1 mg in 100 mL of normal saline. The patient blood urea and creatinine levels were monitored as the drug can cause renal toxicity. And the patient also underwent sinonasal debridement during surgical management and the healing was satisfactory in followup.

## 3. Discussion

Mucormycosis is the third invasive mycosis in order of importance after candidiasis and aspergillosis and is caused by fungi of the class Zygomycetes [4]. The most important species in order of frequency is *Rhizopus arrhizus* (oryzae) [4]. Based on clinical presentation and the involvement of a particular anatomic site, mucormycosis can be divided into at least six clinical categories: (i) rhinocerebral, (ii) pulmonary, (iii) cutaneous, (iv) gastrointestinal, (v) disseminated, and (iv) miscellaneous. Chakrabarti et al. observed that rhino-orbito-cerebral type (44.2%) was the commonest presentation followed by cutaneous (15.5%) and renal (14.0%) involvement in their retrospective analysis for ten years in India [5]. The rhinomaxillary form of the disease, a subdivision of the rhinocerebral form, begins with the inhalation of the fungus by a susceptible individual [6]. Once the spores have penetrated the lungs or subcutaneous tissues, they are met by the first line of defence, mononuclear and polynuclear phagocytes [4]. The phagocytes of the healthy host are able to kill the spores of Mucorales by generating oxidative metabolites and defensins (cationic peptides) [4].

Uncontrolled diabetes mellitus, because of ketoacidosis, can alter the normal immunologic response of patients to infections [7]. Such patients have decreased granulocyte phagocytic ability with altered polymorphonuclear leukocyte response [7]. In diabetic patients *Rhizopus arrhizus* produce the enzyme ketoreductase, which allows them to utilize the patient's ketone bodies [8]. The increased risk of mucormycosis in patients with ketoacidosis may also be due to the release of iron bound to proteins [4].

Fungal invasion of oronasal cavity or paranasal sinuses of susceptible host causes consistent symptoms, sinusitis or periorbital cellulitis, and facial numbness, followed by the onset of conjunctival suffusion, blurry vision, and soft tissue swelling followed by eschar formation and necrosis of nasofacial region [9, 10]. Advancing infection usually spreads from the ethmoid sinus to the orbit, resulting in loss of extraocular muscle function and proptosis with marked chemosis and can quickly result in cavernous sinus thrombosis, carotid artery, or jugular vein thrombosis (Lemierre syndrome) and death [9, 10]. A clinical suspicion of mucormycosis requires confirmation by radiological examination, preferably a CT scan of the maxilla and orbit, showing membrane or periosteal thickening and bony disruption [8]. Imaging findings may be nonspecific and include unilateral or bilateral pan sinus inflammatory changes such as polypoid mucosal thickening. Foci of hyperdensity in the affected sinus on CT scans are highly suggestive of fungal disease [11]. CT is 100% sensitive and 78% specific in the diagnosis of sinonasal mycosis [12]. The phycomycetes and *Aspergillus* are the most common opportunistic pathogens in immunocompromised patients [13]. So we should differentiate aspergillosis from mucormycosis. Radiographically, aspergillosis shows radiological concretions whereas opacification is seen in case of mucormycosis as also being seen in the present case [13]. The diagnosis is made by biopsy of infected tissues. The biopsy should demonstrate the characteristic wide, ribbon-like, aseptate hyphal elements that branch at right angles. The organisms are often surrounded by extensive necrotic debris.

Four factors are critical for eradicating mucormycosis: rapidity of diagnosis, reversal of the underlying predisposing factors (if possible), appropriate surgical debridement of infected tissue, and appropriate antifungal therapy [9].

When diagnosed early, mucormycosis may be cured by a combination of surgical debridement of the infected area and systemic administration of amphotericin B for up to 3 months [6]. Proper management of the underlying disorder is an important aspect affecting the final outcome of treatment [6]. Hyperbaric oxygen therapy has also been used to treat mucormycosis.

Prognosis involves high morbidity and mortality and may improve with rapid diagnosis, early management, and reversible underlying risk factors [14]. Survival rates among groups of patients with invasive sinus disease without cerebral involvement may be as high as 50–80%; if infection spreads to the brain, case fatality ratios exceed 80% [14]. The authors propose that oral physician should be aware of the novel perspectives of the disease for early diagnosis and management.
